# Grafting TRAIL through Either Amino or Carboxylic Groups onto Maghemite Nanoparticles: Influence on Pro-Apoptotic Efficiency

**DOI:** 10.3390/nano11020502

**Published:** 2021-02-17

**Authors:** Hanene Belkahla, Andrei Alexandru Constantinescu, Tijani Gharbi, Florent Barbault, Alexandre Chevillot-Biraud, Philippe Decorse, Olivier Micheau, Miryana Hémadi, Souad Ammar

**Affiliations:** 1 Université de Paris, CNRS-UMR 7086, Interfaces, Traitements, Organisation et DYnamique des Systèmes (ITODYS), UFR de Chimie, 15 rue Jean-Antoine de Baïf, 75013 Paris, France; hanen_belkahla@hotmail.fr (H.B.); florent.barbault@univ-paris-diderot.fr (F.B.); alexandre.chevillot@univ-paris-diderot.fr (A.C.-B.); philippe.decorse@univ-paris-diderot.fr (P.D.); 2 Lipides Nutrition Cancer, INSERM-UMR 1231, Université de Bourgogne Franche-Comté, UFR Science de Santé, 7 Bd Jeanne d’Arc, 21000 Dijon, France; andrei.ac@windowslive.com (A.A.C.); olivier.micheau@inserm.fr (O.M.); 3 Nanomedicine, Imagery and Therapeutics, EA 4662, Université de Bourgogne Franche-Comté, UFR Sciences & Techniques, 16 Route de Gray, 25030 Besançon CEDEX, France; tijani.gharbi@me.com

**Keywords:** surface functionalization, maghemite, cell viability, cancer disease, molecular modeling

## Abstract

Tumor necrosis factor (TNF)-related apoptosis-inducing ligand (TRAIL) is a member of the TNF cytokine superfamily. TRAIL is able to induce apoptosis through engagement of its death receptors DR4 and DR5 in a wide variety of tumor cells while sparing vital normal cells. This makes it a promising agent for cancer therapy. Here, we present two different ways of covalently grafting TRAIL onto maghemite nanoparticles (NPs): (a) by using carboxylic acid groups of the protein to graft it onto maghemite NPs previously functionalized with amino groups, and (b) by using the amino functions of the protein to graft it onto NPs functionalized with carboxylic acid groups. The two resulting nanovectors, NH-TRAIL@NPs-CO and CO-TRAIL@NPs-NH, were thoroughly characterized. Biological studies performed on human breast and lung carcinoma cells (MDA-MB-231 and H1703 cell lines) established these nanovectors are potential agents for cancer therapy. The pro-apoptotic effect is somewhat greater for CO-TRAIL@NPs-NH than NH-TRAIL@NPs-CO, as evidenced by viability studies and apoptosis analysis. A computational study indicated that regardless of whether TRAIL is attached to NPs through an acid or an amino group, DR4 recognition is not affected in either case.

## 1. Introduction

**Figure 1:**
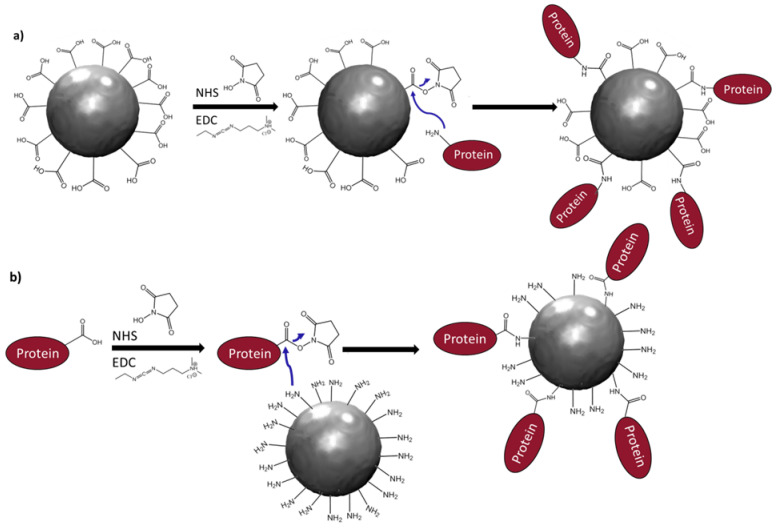
Schematic representation of protein–nanoparticle (NP) coupling based on the EDC–NHS route, activating the NP (**a**) or the protein (**b**) carboxylic groups.

The tumor necrosis factor (TNF)-related apoptosis-inducing ligand (TRAIL) is a member of the TNF family that selectively induces cancer cell death by apoptosis. TRAIL-induced apoptosis is mediated by the transmembrane receptors death receptor DR4 (also known as TRAIL-R1) and DR5 (TRAIL-R2) and requires the accumulation and aggregation of TRAIL and its receptor on the membrane of the malignant cell [1]. It has been demonstrated that conjugating TRAIL with a nanometer-sized, biocompatible, and rigid carrier allows such an aggregation, significantly improving its pro-apoptotic effect [2,3,4,5]. To date, several carriers have been tested with special emphasis on their covalent bonding to TRAIL. Indeed, a prerequisite for advancing this area of research is the development of chemical methods to conjugate the ligand in a reliable manner with a nanomaterial, the carrier, which may be inorganic (such as silica, gold, and iron oxide) or organic (such as latexes, liposomes, and carbon dots). Several chemical methods have been developed over the past decades to synthesize such nanovectors by focusing on (i) strong bonding between TRAIL and nanoparticles (NPs) and (ii) stable biological activity [4]. This means that the in vivo administration of the engineered hybrids must not alter the constructs by breaking chemical bonds or changing their affinities for the biological targets. As a protein, TRAIL has terminal carboxylic acid and amino groups which can be activated to attach onto the surface of NPs. While this opens up several routes for grafting and surface modification of nanoparticles, TRAIL grafting has, until now, always been performed using its amino acid terminal groups, to preserve its binding capacity. Strategies where the grafting of TRAIL was notably involved include (a) the glutaraldehyde reaction with TRAIL, resulting in alkylation of available amino functions at around neutral pH, forming stable secondary amine bonds [4]; (b) the reductive amination by sodium cyanoborohydride of the terminal amino group to react with NP surface aldehyde groups [6]; (c) peptide coupling where carboxylic groups (of TRAIL or NPs) are activated before their reaction with amino groups (of NPs or TRAIL) [3,5]. In nanomedicine, the latter route is called the EDC–NHS route (1-ethyl-3-(3-dimethylaminopropyl)carbodiimide (EDC)–N-hydroxysuccinimide (NHS)) [7]. It is commonly used to couple a protein to a particle surface [8,9,10,11]. In practice, the carboxylic acid is transformed into a reactive ester which then reacts with an amino group, as illustrated in Figure 1a.

In this study, we compare the two associations of TRAIL and NPs, namely activating the carboxylic groups of TRAIL or those of the NPs, working on a specific system of 10 nm single crystals of magnetic iron oxide produced by the well-known polyol method [12,13]. Magnetic nanocrystals with particle sizes of about 10–30 nm combine superparamagnetic behavior [14] with a saturation magnetization close to the bulk value and smaller saturating fields [15], making them particularly valuable for magnetically assisted biomedical applications. The main idea is to construct a magnetically stimulable antitumoral agent, taking advantage of the properties of its components to combine malignant cell targeting, the apoptotic activity of TRAIL, magnetic resonance imaging (MRI), and/or magnetic hyperthermia (MH) by the NPs. We already established that these bare iron oxide NPs exhibit intrinsically good magnetic properties suitable for MRI [16] and MH [17] as efficient negative contrast agents and heating probes, respectively. We have recently demonstrated that their optical properties are suitable for use in photothermal therapy, increasing TRAIL antitumoral properties [3] as efficiently as mild hyperthermia [18]. An advantage of using iron oxide NPs is their lack of cytotoxicity for non-malignant cells at low concentration [10]. Moreover, under these conditions they are readily degraded in cells and in vivo at the low pH of endosomes, and iron homeostasis is assured by the iron-storage protein, ferritin [19]. This makes such NPs very appealing for therapeutic applications. Magnetite NPs were functionalized with two different linkers, citrate [20] and 3-aminopropyltriethyloxysilane (APTES) [8], which introduce carboxylic acid (Figure 1a) and amino groups (Figure 1b), respectively. The resulting nanohybrids are designated as NPs-CO and NPs-NH, respectively. TRAIL was then grafted onto these nanohybrids, to give the nanovectors, NH-TRAIL@NPs-CO and CO-TRAIL@NPs-NH. Characterization techniques, including SQUID magnetometry, X-ray photoelectron spectroscopy (XPS), transmission electron microscopy (TEM), Fourier transform infrared spectroscopy (FTIR), and dynamic light scattering (DLS), were used to evaluate their properties and to determine their grafting efficiency. Biological studies were performed on human breast and lung carcinoma cells (MDA-MB-231 and H1703 cell lines) in order to establish that nanovectors are potential agents for cancer therapy. A computational study was also performed to see whether there is a preference for TRAIL to be attached to NPs through acid or amino groups, which might explain any difference in the pro-apoptotic effects of the two nanovectors. The originality of this work lies in establishing that TRAIL can be grafted onto iron oxide NPs by either its carboxylic or its amino groups without losing its targeting capabilities and pro-apoptotic efficiency.

## 2. Materials and Methods

### 2.1. Materials

All chemicals were of the purest commercial grade. Iron acetate (Fe(CH_3_CO_2_)_2_) (Acros (Molinons, France), 95%), diethyleneglycol (DEG) (Merck, ReagentPlus^®^(Paris, France), 99%), APTES (Aldrich (Paris, France), 99%), sodium citrate (Sigma-Aldrich (Lyon, France), 99%), and ethanol, methanol, paraformaldehyde (PFA), phosphate-buffered saline (PBS), 2-(N-morpholino)ethanesulfonic acid (MES) buffer, methylene blue, N-hydroxysuccinamide (NHS), and 1-ethyl-3-(3-dimethylaminopropyl)carbodiamide (EDC), all from Merck (Paris, France), were used as received.

### 2.2. Maghemite Nanoparticle (NP) Synthesis

**Figure 2:**
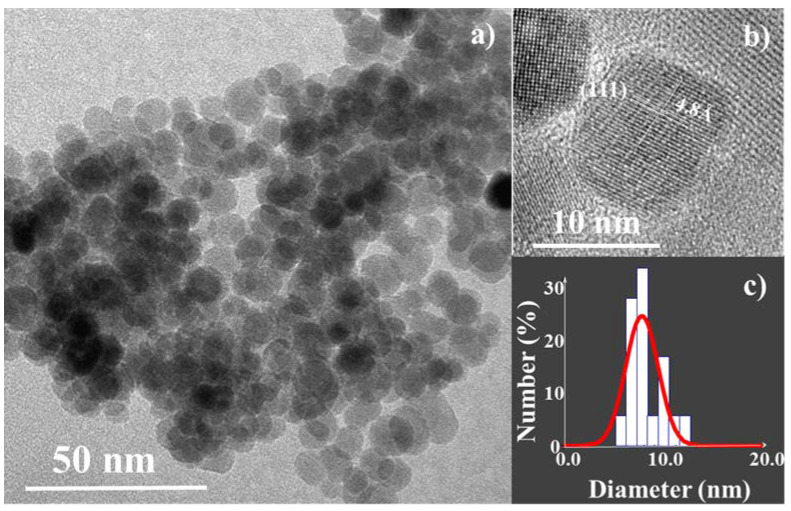
(**a**) TEM image of a group of iron oxide NPs. (**b**) Inset: HRTEM image of one representative NP showing interplanar distances of 4.8 Å corresponding to the maghemite (111) atomic planes [21]. (**c**) Size distribution.

Almost monodisperse 10 nm maghemite (γ-Fe_2_O_3_) NPs (Figure 2) were synthesized by forced hydrolysis of iron acetate in DEG [9]. Distilled water (1 mL) and 4.3 g of ferrous acetate, Fe(CH_3_CO_2_)_2_, were dissolved in 250 mL of DEG under mechanical stirring. The mixture was heated (6 °C min^−1^) to the boiling point (230 °C) and maintained under reflux for 3 h before cooling to room temperature. The precipitated magnetic powder was then recovered by centrifugation, washed with hot water twice, and finally dried at 50 °C for several hours. Its structure and microstructure were checked by transmission electron microscopy (TEM) using a JEOL-100-CX II (JEOL, Tokyo, Japan) microscope operating at 100 kV.

### 2.3. Elaboration of Magnetic Nanohybrids

Maghemite NPs were functionalized by treatment with APTES or sodium citrate. TRAIL was produced and used as described previously [22]. Sol–gel chemistry and surface complexation were used for the silane- and citrate-treated NPs, respectively. Amino nanoparticles (NPs-NH) were obtained by dispersing 100 mg of as-produced maghemite NPs in 4 mL of methanol under sonication for a few minutes. The suspension was then diluted in 100 mL of ethanol, and 1 mL of APTES was added. The reaction solution was mechanically stirred for 1 h at room temperature and then heated to boiling for 2 h under argon atmosphere. Citrate nanoparticles (NPs-CO) were obtained by dispersing 1 g of as-produced maghemite NPs in 200 mL of an aqueous sodium citrate solution (50 mM). The suspension was mechanically stirred and heated at 100 °C for 30 min. In both cases, the resulting hybrids were collected by a magnet, washed with an excess of methanol (for NPs-NH) or ethanol (for NPs-CO), to remove excess reactant and dried in air at 50 °C overnight.

### 2.4. Elaboration of Nanovectors

CO-TRAIL@NPs-NH was prepared by the following steps: To 500 μL of a solution of TRAIL (1 mg mL^−1^) in PBS buffer at pH 7, microvolumes of NHS (final concentration: 3.75 mM) and EDC (final concentration: 1.5 mM) were added. The mixture was stirred for 1 h at room temperature. NPs-NH (5 mg) was dispersed in 1 mL of PBS buffer at 8.5 ≤ pH ≤ 9 and then added to the protein mixture. The suspension was stirred at 4 °C overnight. The suspension was washed several times with PBS buffer (pH 7) to remove the remaining NHS ester and the excess of TRAIL. CO-TRAIL@NPs-NH was recovered by decantation on a laboratory magnet.

To produce NH-TRAIL@NPs-CO, 5 mg of NPs-CO were dispersed in 1 mL MES buffer at pH 6, and microvolumes of NHS (final concentration: 3.75 mM) and EDC (final concentration: 1.5 mM) were added. The mixture was stirred for 1 h at room temperature. 500 μL of a solution of TRAIL (1 mg mL^−1^) in 1 mL of PBS buffer at 8.5 ≤ pH ≤ 9 were added to the nanoparticle dispersion. The mixture was stirred at 4 °C overnight, then washed several times and the nanovector NH-TRAIL@NPs-CO was collected on a laboratory magnet.

Protein concentrations in the nanovectors were determined on a Cary 4000 spectrophotometer (Agilent, Les Ulis, France) by measuring the absorbance at 280 nm and with Bio-Rad protein assay by measuring the absorbance at 595 nm [23]. The iron concentration in the nanovectors was determined by Prussian blue assay [3].

### 2.5. Characterization Techniques

The functionalization of NPs with citrate and silane as well as the reaction of the resulting hybrids with TRAIL were monitored using several characterization techniques.

FTIR spectra for NPs, nanohybrids, and nanovectors were acquired by the KBr technique on a Bruker Equinox spectrometer (Bruker, Billerica, MA, USA) working in the 500-4000 cm^−1^ range with a resolution of 4 cm^−1^.

X-ray photoelectron spectroscopy (XPS) was performed to check the chemical composition of the surface of the bare NPs, hybrids, and nanovectors. A Thermo VG ESCALAB 250 instrument (Thermo Fisher Scientific, East Grinstead, UK) equipped with a microfocused, monochromatic Al Kα X-ray source (1486.6 eV) and a magnetic lens was used. The X-ray spot size was 650 µm (15 kV, 150 W). The spectra were acquired in the constant analyzer energy mode with pass energies of 150 and 40 eV for the general survey and the narrow scans, respectively.

Thermogravimetric (TG) analysis was conducted in air on a Labsys-Evo (Setaram Instrumentation, Caluire-et-Cuire, France) with a heating rate of 10 °C min^−1^, up to 600 °C, to quantify the organic content of the hybrids and nanovectors.

The surface charges of bare NPs, hybrids, and nanovectors were determined on colloidal aqueous solutions (1 g L^−1^), vigorously sonicated (10 min) beforehand, at room temperature, using a Malvern Nano Zetasizer (Malvern, Worcestershine, UK)). The hydrodynamic diameter was determined on the same equipment by dynamic light scattering (DLS) in water.

The magnetic properties of maghemite nanoparticles (NPs) and their related hybrids and nanovectors were inferred from field-dependent magnetization curves recorded on a Quantum Design MPMS-5S SQUID magnetometer (Quantum Design, San Diego, CA, USA) at 5 and 310 K. The external magnetic field was varied from −70 to +70 kOe. Temperature-dependent magnetization plots were also recorded at low field (0.2 kOe) at temperatures from 5 to 350 K in the field-cooled (FC) and zero-field-cooled (ZFC) modes. The powders were slightly compacted in a diamagnetic plastic sampling tube to avoid their displacement during measurements.

### 2.6. Cell Lines and Cell Culture: Viability Assay and Apoptosis Survey

Human breast carcinoma (MDA-MB-231) and lung carcinoma (H1703) cells were purchased from the American Type Culture Collection (Manassas, USA). They were maintained in Dulbecco’s Modified Eagle’s medium (DMEM) and the Roswell Park Memorial Institute (RPMI) medium (Lonza, Levallois-Perret, France) supplemented with 10% and 5% fetal calf serum, respectively. Cells were cultured in 5% CO_2_.

Viability of these cells was assessed by methylene blue assay. Cells (5 × 10^4^) were seeded per well (0.32 cm^2^) in 96-well plates and incubated as above with bare NPs or the nanovectors at concentrations from 0 to 10^4^ ng mL^−1^ at 37 °C.

After 16 h, cells were washed in cold PBS buffer solution and fixed by 2% PFA for 20 min at room temperature, washed three times in PBS, and stained with 5% methylene blue for 30 min. After three subsequent gentle washes in PBS, methylene blue was eluted in 1% HCl for 4 h at ambient temperature. Optical density was then measured at 630 nm.

Cell apoptosis was determined by flow cytometry for annexin V and propidium iodide detection. Cells were labeled using the APC Annexin V (a5) Apoptosis Detection Kit with propidium iodide (PI) (Biolegend). Cells were detached (100,000 cells per condition), washed twice in PBS buffer, suspended in 100 μL of binding buffer to which a5 (5 μL), and PI (10 μL) were added. After 15 min of incubation, 400 μL of binding buffer were added, and cells were analyzed on a flow cytometer (BD FACSCanto™ 10 platform, BD Biosciences, Le Pont de Claix, France). Apoptosis is expressed as the percentage of cells presenting positive labeling vs. negative-labeled counterparts (viable cells: a5 − & PI −; early apoptosis: a5 + & PI −; late apoptosis/necrosis a5 + & PI +; debris: a5 − & PI + etc.). Each statistical analysis is based on a minimum of 10,000 events. For each condition, the number of independent measurements was systematically greater than 3 (*n* > 3). Three independent cell preparations were performed for each method. Values are expressed as the mean ± standard error of the mean.

### 2.7. Computational Study

The TRAIL fragment was constructed from the reference protein data bank (pdb) structure 5CIR [24]. The missing residues were predicted using the i-tasser web server [25] and the amino acid charges were assigned using the H++ web server according to electrostatic computations at physiological pH [26]. The resulting protein model was immersed in a water solvent box and subjected to a molecular dynamics study for 50 ns at 300 K with the Amber software [27]. Details on this last procedure may be found elsewhere [28] and were used to obtain more reliable structural results and information on residue flexibility. To estimate the accessibility of DR4 to TRAIL when the latter is grafted onto a NP, a spherical 5 nm radius iron oxide NP was created with the Avogadro software [29] by extending the maghemite crystal structure in 3 dimensions to obtain a rectangular box with its lowest dimension greater than 10 nm. Structural analyses and visualizations were achieved with VMD [30] and cpptraj [31] software.

## 3. Results and Discussion

### 3.1. Structural and Microstructural Properties

**Figure 3:**
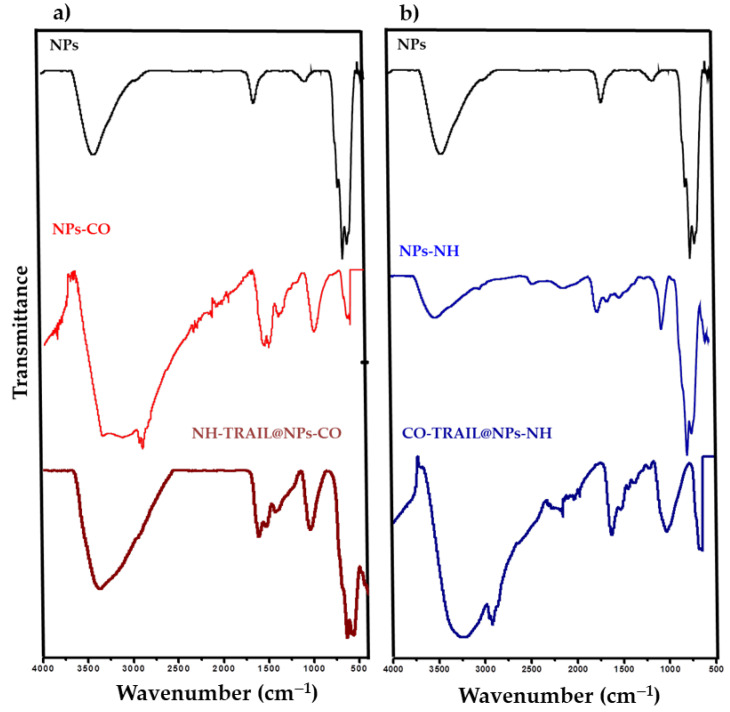
FTIR spectra of NPs-CO (**a**) and NPs-NH (**b**) compared to that of bare NPs. Spectra of their related TRAIL nanovectors are also given.

Figure 3 reports the IR spectra for the citrate and silane functionalizations. The absorption bands in all spectra at 634 and 579 cm^−1^ are typical of Fe–O in maghemite [32]. For NPs (Figure 3, black), the bands at 1640 and 3420 cm^−1^ are those of δOH and νOH (water), respectively. The intensity of carboxylate vibration bands between 1600 and 1400 cm^−1^ increases after citrate treatment and also after TRAIL coupling (Figure 3, red). A Si–O vibration band appears at about 1050 cm^−1^ after silane treatment in both NPs-NH and CO-TRAIL@NPs-NH samples (Figure 3, blue). The intensities of the hydroxyl (O–H) and amino (N–H) vibration bands at around 3300 cm^−1^ are enhanced in the spectra of TRAIL-based vectors (Figure 3, dark blue and red). The band at 1615 cm^−1^ is assigned to amide I (C=O stretching) and is related to the secondary structure of TRAIL (α helix) [33]. The 1538 cm^−1^ band is assigned to amide II (CN stretching and NH bending). These bands establish the presence of TRAIL at the surface of NPs.

Transmission electron microscopy (TEM) and dynamic light scattering (DLS) were performed to measure the average crystal size, <D_TEM_>, and the hydrodynamic diameter, <D_DLS_>, of all the nano-objects dispersed in water. While all the particles, bare and hybrids, exhibit the same <D_TEM_> = 10 nm, their <D_DLS_> values change from one sample to another. Typically, bare NPs are slightly aggregated, whereas citrate and silane surface-modified ones are more stable and have <D_DLS_> values of 12 and 34 nm, respectively (Figure S1). This suggests the production of individual functionalized particles in both cases with, however, larger APTES-based NPs, which may be due to the condensation of silane molecules at their surface [34].

The ζ potentials of all these particles were measured at room temperature, by varying the pH of their aqueous solutions. As expected, there is a net variation of the surface charge between bare NPs and silane or citrate surface-modified NPs and their TRAIL-grafted counterparts. The isoelectric point (IEP) of bare NPs is pH 7.7. It falls to pH 2.2 and rises to pH 8.9 when they are coated by acid and amino groups, respectively, in agreement with the negative and positive surface charges expected under physiological conditions (Figure S1). Interestingly, TRAIL grafting reduces this difference, with a shift of the former IEP values to the same value, pH 6.6 (not shown), close to that of free TRAIL, pH 7.0. [34] Clearly, the citrate and silane coatings not only serve as a platform for TRAIL functionalization but also act as hydrophilic layers to provide colloidal stability, avoid aggregation, and prevent any change of the original structure of the NPs.

**Figure 4:**
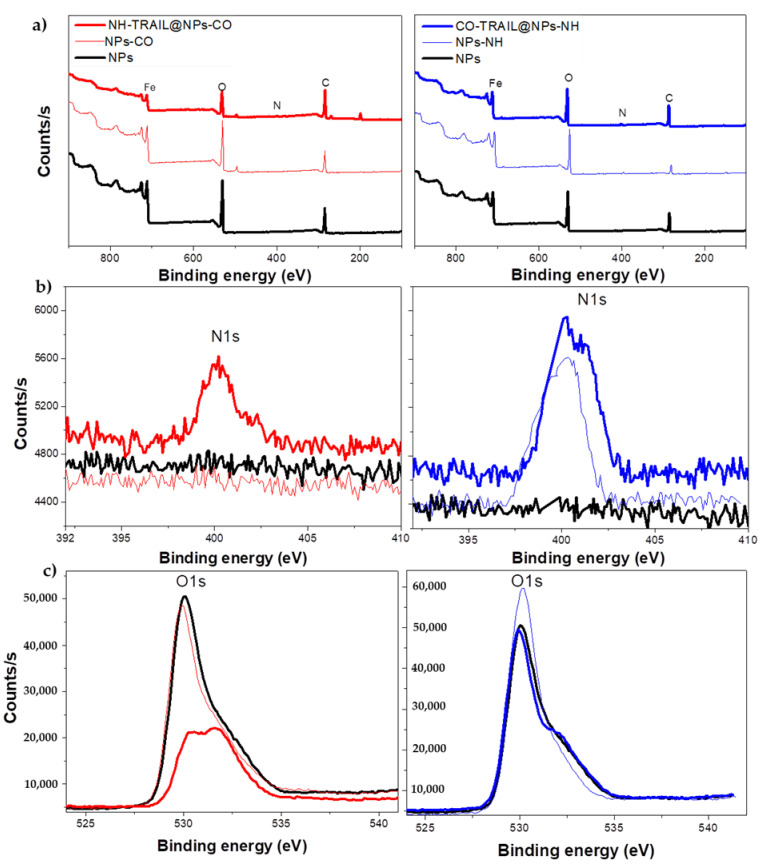
(**a**) XPS survey and high-resolution N1s (**b**) and O1s (**c**) spectra: bare NPs (black), NPs-CO (light red), NPs-NH (light blue) hybrids, and the related nanovectors NH-TRAIL@NPs-CO (red) and CO-TRAIL@NPs-NH (blue).

These measurements were completed by X-ray photoelectron spectroscopy (XPS) analysis to focus on the chemical surface composition of the materials. All the spectra evidence Fe, C, and O atoms, with N atoms being observed only in NH-TRAIL@NPs-CO, CO-TRAIL@NPs-NH and NPs-NH (Figure 4). The increase in the O/Fe and N/Fe atomic ratios agrees with TRAIL grafting (Table 1).

The high-resolution O 1s spectrum of bare NPs exhibits a main peak at 530 eV attributed to the spinel lattice contribution [35], while those of the TRAIL-based nanovectors show an additional contribution at 533 eV attributed to carbonyl groups, including those involved in the amido group formed upon coupling [36]. The intensity of this last contribution increases from NPs to NPs-CO and NH-TRAIL@NPs-CO, and from NPs to NPs-NH and CO-TRAIL@NPs-NH, the surface layer being increasingly made up of the organic coating rather than by iron oxide.

There is no signal in the high-resolution N 1 s spectrum of bare NPs, meaning that nitrogen species are absent from the surface of the maghemite particles. The spectra of TRAIL-based nanovectors show a broad, asymmetric signal, centered at 399.9 and 401.6 eV (Figure 4). These binding energies are usually assigned to non-protonated/protonated amino and amido species, respectively, present in the protein [37]. Moreover, the relative amino and amido peak intensities indicate that the amino peak always predominates in the nanovectors. This feature means that a non-negligible part of the TRAIL amino/ammonium groups remains accessible after TRAIL coupling to NPs. This point must be underlined because if such groups contribute to ligand–receptor recognition, our XPS analysis shows that there are still unreacted ones available.

**Figure 5:**
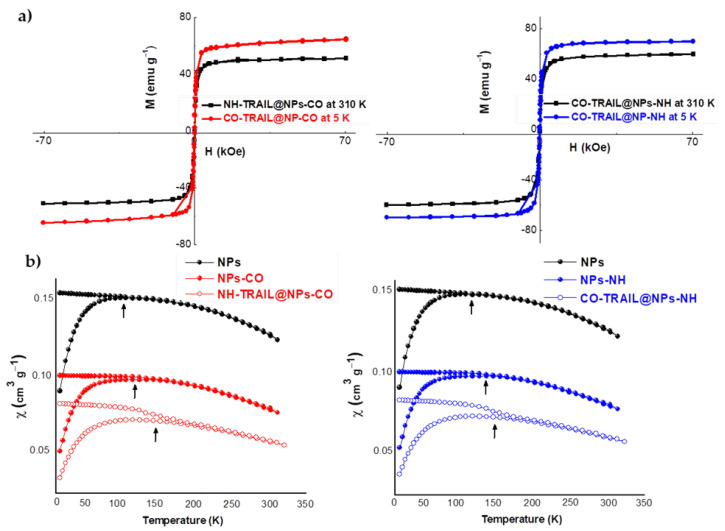
(**a**) Hysteresis loops recorded at 310 and 5 K on NP-CO_2_H-TRAIL and NP-NH_2_-TRAIL hybrids. (**b**) Thermal variation of the DC magnetic susceptibility of bare NPs, nanohybrids, and the related NH-TRAIL@NPs-CO and CO-TRAIL@NPs-NH nanovectors, measured at 200 Oe on as-produced powders. The arrows indicate the blocking temperature (BT).

The surface coverage of NPs by TRAIL was estimated by different techniques. We first evaluated the weight content of grafted TRAIL by TG and magnetometry analyses, and then compared it to that inferred from Bio-Rad assay experiments. TG results on NH-TRAIL@NPs-CO and CO-TRAIL@NPs-NH (Figure S2, Supplementary Information) show a significant weight loss above 100 °C, assumed to be due to the departure of chemisorbed organic species, mainly TRAIL, and their decomposition (below 100 °C, only physisorbed water departs). The measured weight loss in these samples is significantly higher than that for bare NPs and still higher than for NPs-CO and NPs-NH. The total weight loss for NH-TRAIL@NPs-CO and CO-TRAIL@NPs-NH subtracted from that for NPs-CO and NPs-NH, respectively, is consistent with TRAIL contents of about 15.8 and 13.0 wt%, respectively (Table 2). The drop in the saturation magnetization on going from NPs-CO or NPs-NH to NH-TRAIL@NPs-CO or CO-TRAIL@NPs-NH, respectively (see the 5 K hysteresis loops in Figure 5), also gives an estimate of the TRAIL weight content (Table 2). The magnetometry values are very close to those obtained by TG analysis and Bio-Rad assay. The difference between the values for the citrate and the silane systems is small, suggesting that whatever the NP and the nature of the linker, the amount of TRAIL attached is almost the same, making both peptide coupling procedures (Figure 1) valid for its conjugation.

### 3.2. Magnetic Properties

The thermal variations in the magnetic susceptibility χ(T) of the powder samples were measured in both zero-field-cooling (ZFC) and field-cooling (FC) conditions. The χ(T) curves for bare NP and its related hybrids are shown in Figure 5. A net irreversibility between the ZFC and FC branches is observed: the ZFC plots show a clear maximum at a critical temperature defined as the blocking temperature (BT), in agreement with the usual results [38,39]. BT represents the threshold temperature above which the magnetic anisotropy barrier is overcome solely by thermal activation energy, causing the NPs to relax from the ferrimagnetic to the superparamagnetic state. These trends in ZFC and FC are typical of superparamagnetism in magnetic single domains. The average BT values, estimated from the magnetic susceptibility, increase from 120 K to about 150 K when the aggregation state of Fe_2_O_3_ nanocrystals decreases, namely from bare NPs to NPs coated with silane or citrate, and then TRAIL conjugates. This behavior means that the weakening of the interparticle interaction is completely consistent with Stoner–Wohlfarth theory [40]. It has already been reported [41] that the FC-χ(T) curve for non-interacting superparamagnets continues to rise with a decrease in temperature below BT, and a deviation from this variation is mainly due to interparticle interactions. These reports confirm that dipole–dipole interactions occur in our systems but are weaker when TRAIL is present. These interactions could affect the magnetocalorimetric properties of magnetic single domains for eventual application in magnetic hyperthermia [16].

### 3.3. Antitumoral Properties and Capabilities of the Nanovectors in Cancer Therapy

For all the biological assays, the tested TRAIL and nanovectors doses are expressed as ligand concentrations in ng mL^−1^. In the case of nanovectors, they correspond to the amount of grafted TRAIL per mL of hybrid solution, while for NPs, they correspond to the same iron oxide content ([Fe] = 15 mM) as in their nanovector counterpart solutions.

**Figure 6:**
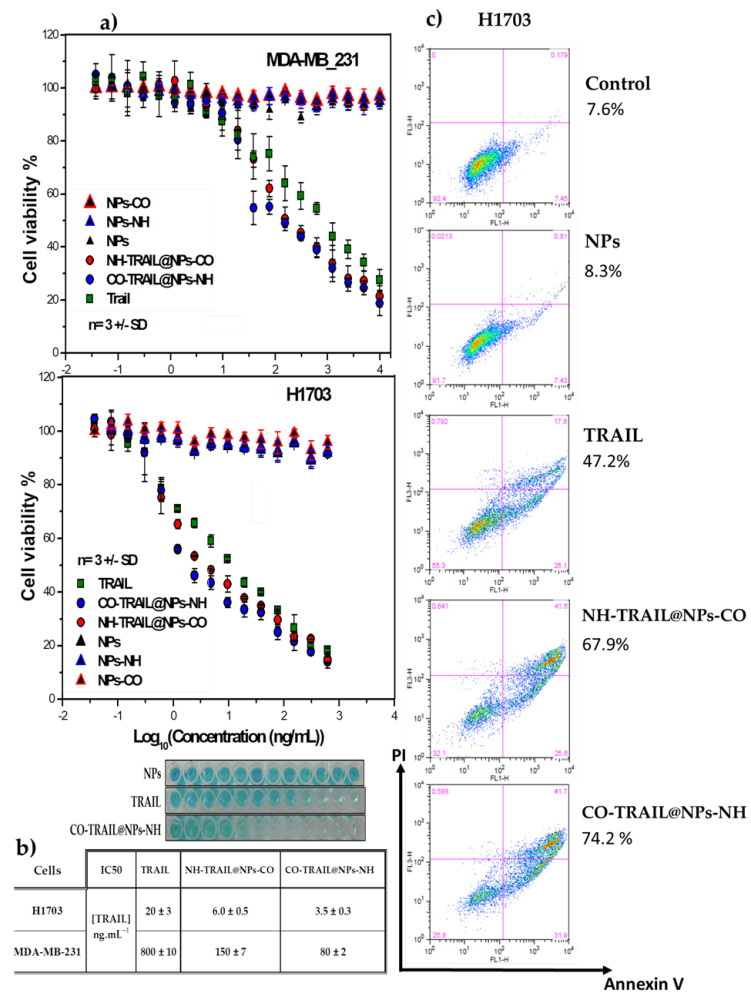
(**a**) Cell death induced by TRAIL and CO-TRAIL@NPs-NH and NH-TRAIL@NPs-CO nanovectors in MDA-MB-231 and H1703 cells. Methylene blue staining was used to measure cell viability with increasing concentrations of indicated compounds. (**b**) IC50 values expressed as TRAIL concentration. (**c**) Early and late apoptosis and/or necrosis were determined by a5 and PI staining of H1703 cells treated by TRAIL, NPs, and the nanovectors. The indicated percent represents cells positive for both markers and was calculated by FlowJo software after flow cytometry.

NPs alone and nanohybrids without TRAIL showed no deleterious effect on MDA-MB-231 or on H1703 cell lines (Figure 6). Nanovectors induce a dose-dependent degradation in cell viability with a median lethal concentration (IC50) ranging between 3 and 6 ng mL^−1^ for H1703 and between 80 and 150 ng mL^−1^ for MDA-MB-231, substantially lower than for TRAIL alone (IC50 approx. 20 and 800 ng mL^−1^, respectively), as summarized in Figure 6b. Using annexin-V/PI, we established that NH-TRAIL@NPs-CO and CO-TRAIL@NPs-NH induce apoptosis with a significantly greater efficiency than TRAIL. Apoptosis is highly enhanced by NH-TRAIL@NPs-CO (67.9 ± 3.5)% and CO-TRAIL@NPs-NH (74.2 ± 2.5)% stimulation compared to free TRAIL (47.2%) or bare NPs (8.3%), as illustrated in Figure 6 for the H1703 cell line.

Targeting is an important issue in nanomedicine. The aim is to build specific vectors, passive or active, able to deliver the desired dose of a drug to diseased biological tissues. Good targeting has to be efficient, safe, meet clinical requirements and, above all, be ‘druggable’. A ‘druggable’ target is accessible to the putative drug molecule, be it a small molecule or larger biological, and when the vector binds, it must elicit a biological response which is measurable both in vitro and in vivo. TRAIL respects all these requirements. It may act simultaneously as a drug and as a targeting substrate since it is able to induce malignant cell apoptosis through its specific bonding with DR4 and DR5. The nanovectors are safe for cells which do not express these receptors, namely most healthy cells. Attaching TRAIL to a solid carrier like a magnetic particle increases its solubility in the physiological media and, therefore, its lifetime in the blood circulation. It also improves its pro-apoptotic effect, since its concentration on the surface of a single nanometer-sized object leads to the simultaneous delivery of a large number of TRAIL molecules to all accessible malignant cells.

Given that a primary desirable characteristic of drugs is that they should be effective at low concentrations, the decrease in the IC50 values for two stimulated human carcinoma cell models on going from free TRAIL and TRAIL attached to NPs indicates that the nanovectors should also perform in vivo. A second major challenge is the stability of the nanovectors. In other words, TRAIL must not dissociate from the iron oxide particle surface under in vitro or in vivo conditions. Our preliminary biological assays tend to confirm that the nanovectors are stable and that the protein is covalently bonded to the NP.

A third challenge is that the grafting of TRAIL onto the nanohybrids should not affect its interaction with the death receptors. These results indicate that the nanovectors respect this condition, even if that based on the amino platform is slightly more potent than that obtained using acid residues. The IC50 values for model tumoral human cells using CO-TRAIL@NPs-NH and NH-TRAIL@NPs-CO are significantly smaller than those for free TRAIL, but those for the former are about half those for the latter. Moreover, we recently demonstrated the synergistic effect of TRAIL grafted onto nanoclusters and combined with magnetic hyperthermia or photothermia in increasing cancer cell death. TRAIL-based nanovectors are amongst the most promising for the treatment of cancer [3]. A fourth criterion is that the delivery of the nanovector to the tumor in the body should be selective. An enhanced permeability and retention effect can be used as a complementary strategy to active targeting. The active and passive targeting capabilities of the TRAIL-based nanovector contribute to enhancing its chance of success in clinical assays [42].

### 3.4. Computational Study on TRAIL–DR4 Recognition

The main idea is to check whether there is any perturbation in TRAIL–DR4 recognition when TRAIL is attached to NPs through a carboxylic or an amino group. To this end, a computational protocol was employed to predict the structure of the ligand complex, using crystallographic data on the TRAIL and TRAIL–DR4 complex structures [24].

**Figure 7:**
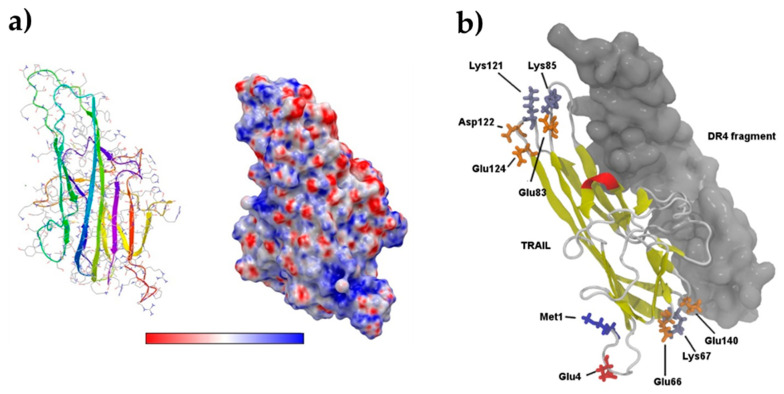
(**a**) TRAIL structure (left) and its molecular surface colored with its electrostatic potential (right), ranging from −9.479 (red) to +9.479 (blue) kT/e. (**b**) TRAIL structure, displayed with a cartoon protein representation, complexed with the DR4 fragment (gray molecular surface). The 10 residues highlighted in Table S1 (gray background) are labeled. Residues bearing a carboxylic group are shown in orange, except for Glu4, the first one in Table S1, which is in red. Similarly, all residues bearing an amino group are shown in ice blue, whereas Met1 is displayed in blue.

TRAIL in its monomeric form was considered. The nature of the chemical interaction of NPs with the TRAIL fragments was investigated. At physiological pH, silane- and citrate-coated NPs are positively and negatively charged, respectively. TRAIL may also be charged through its amino acids (Lys, Arg or Asp, Glu). This means that charged NP may be attached to TRAIL by electrostatic interactions. Our calculation shows that whereas 25% of the TRAIL amino acids are charged (11 Lys, 11 Arg, 6 Asp, and 13 Glu) these mainly interact with each other, leading to numerous salt bridges. As a consequence, TRAIL does not present a specifically polar interface, as evidenced in Figure 7a. In the absence of such a polar surface, the hypothesis that the protein is electrostatically attached to the NP can be rejected, and the possibility of covalent bonding by amide bonds is favored.

Depending on the type of NP functionalization, the formation of amide bonds requires an amino group, that of APTES at the surface of NPs, to react with a free TRAIL carboxylic acid moiety (Asp, Glu) (Figure 1a) or, conversely, a TRAIL amino residue (Lys) reacting with the acid species on the NP surface (Figure 1b). After construction of the TRAIL structure, the protonation states of all residues were evaluated by electrostatic computations at physiological pH. The results show that all histidine residues remain in their neutral form and that TRAIL is in a zwitterionic form. Thus, the first residue bears an amino group (Met1) and the last (Gly169) an acid group; these two residues should be added to the list of potential reaction sites.

To predict where an amide bond can be formed, a detailed structural analysis of TRAIL was performed through a 50 ns molecular dynamics trajectory in a water solvent box at physiological pH. Monitoring kinetic, potential, and overall energies along the trajectory as well as the density, pressure, and temperature demonstrates the stability of the TRAIL conformation. The overall form of TRAIL, as well as its secondary structure, is conserved during the simulation, emphasizing the stability of its structure.

When the NP is covered by citric acid, 12 TRAIL residues are available for formation of an amide bond whereas, when the NP is covered by silane, there are 20 likely TRAIL residues (Table S1). As was proposed for the design of suicide inhibitors, the formation of a covalent bond on a protein requires high accessibility to the atoms implicated in the reaction and a notable flexibility to allow the relaxation of protein structure [43]. Therefore, three distinct metrics were computed along the last 40 ns of the trajectory for all residues considered: (i) their whole solvent-accessible surface area (SASA), (ii) the SASAs of their carboxylic and amino groups, and (iii) their root mean square fluctuation (RMSF). All results are compiled in Table S1, which is organized in decreasing order of carboxylic and amino SASAs. This is the most important metric since a high value denotes a high probability of amide bond formation. In this table, there are six residues bearing acid groups presenting a SASA for the COO^−^ atoms over 160 Å^2^, a whole residue SASA over 300 Å^2^, and RMSF over 14 Å. This last value represents the average value of the RMSF per residue. These six amino acids are displayed with a grey background in Table S1 and are shown in Figure 7b. Similarly, there are four residues presenting an amino group with SASA values over 120 Å^2^, residue SASA over 300 Å^2^, and a RMSF over 16 Å. These four residues, able to form a covalent bond when the NP is functionalized with citrate, are shown on the TRAIL structure in Figure 7b.

**Figure 8:**
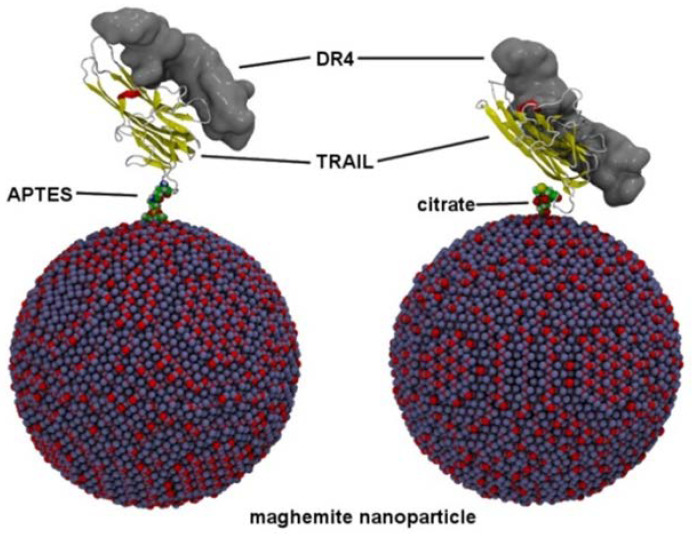
Maghemite NPs were functionalized with APTES (**left**) or citrate (**right**) and covalently bonded to TRAIL Glu4 (**left**) and Met1 (**right**). TRAIL is shown with a cartoon protein representation and DR4 with a gray molecular surface.

In Figure 7b, the positions of the most probable residues implicated in grafting are displayed, and the interaction with the TRAIL fragment is shown. Roughly speaking, if we consider that TRAIL is shaped like a banana, these residues are on the extremities and are remote from the recognition interaction with the DR4 fragment. Moreover, they are on TRAIL loops and not on their secondary structural elements, emphasizing their flexibility, as witnessed by the high RMSF values. Therefore, it can be expected that bonding with TRAIL, either with an amino or a carboxylic group, will not perturb the recognition with the DR4 target and that the functionalized NP will remain biologically active. Within the framework of these simulations, no conclusion can be reached about a difference between silane and citrate functionalization. Structures of two 10 nm diameter maghemite NPs were generated: a molecular extension of silane or citrate and an amide bond with their respective most favorable residues (Met1 and Glu4), neglecting the observed clustering of NPs-NH hybrids (Figure 8). Both Glu4 (Figure 8 left) and Met1 (Figure 8 right) are located significantly far from the recognition site, which means that there is enough room for the metallic NPs not to interfere with DR4 recognition.

At this stage of our theoretical investigations, there is no explanation for the fact that CO-TRAIL@NPs-NH exhibits a higher apoptotic effect than NH-TRAIL@NPs-CO. At this level of calculation, neither strategy for coupling NPs and TRAIL appears to affect the flexibility of the latter, and there should be no effect on receptor recognition. Therefore, it is possible to functionalize TRAIL through either amino or carboxylic groups; this provides more flexibility and opportunities for therapeutic applications.

## 4. Conclusions

Well-crystallized maghemite nanoparticles about 10 nm in diameter were functionalized by silane and citrate groups, making their surfaces positively and negatively charged, respectively, under physiological conditions. The residual amino and carboxylic acid groups form amide bonds with TRAIL through a peptide reaction. Computational studies confirm this finding. This study underlines the fact that structural variations of TRAIL (in other words, the residue flexibility) which occur when it is thus attached to NPs are small, perturbing recognition by the DR4 receptor to a negligible extent.

The TRAIL contents of the nanovectors, determined by Bio-Rad assay, TG, and magnetometry analyses, are of the same order of magnitude, but slightly smaller for CO-TRAIL@NPs-NH than for NH-TRAIL@NPs-CO, about (10 ± 2) and (13 ± 3) mol/particle, respectively. Methylene blue cell viability assays performed on human breast carcinoma (MDA-MB-231) and lung carcinoma (H1703) cells exposed to increasing amounts of free TRAIL, bare NPs, and the two nanovectors evidence an improved apoptotic effect with the last-named. The IC50 value on the resistant MDA-MB-231 cells, using CO-TRAIL@NPs-NH, is a factor of 10 less than that for free TRAIL, but only a factor of 6 less when NH-TRAIL@NPs-CO is used. These results are very interesting and encourage us to engineer other maghemite-based hybrids of TRAIL in the hope of further increasing its pro-apoptotic potential.

## Figures and Tables

**Table 1:** N/Fe and O/Fe atomic ratios inferred from XPS analysis of bare NPs, hybrids, and related nanovectors NH-TRAIL@NPs-CO and CO-TRAIL@NPs-NH.

Ratio	NP	NPs-CO	NH-TRAIL@NP-CO	NPs-NH	CO-TRAIL@NPs-NH
at.-N/Fe	0	0	0.25	0.12	0.20
at.-O/Fe	2.88	2.96	5.59	2.17	3.69

**Table 2:** Quantification of TRAIL grafted on NPs-CO and NPs-NH using different techniques: magnetometry, TG analysis, and Bio-Rad assay. ^(^*^)^ to be compared to 84.3 emu g^−1^ for bare NPs. Data are means ± SD (*n* = 3).

Samples	Magnetometry Analysis			TG		Bio-Rad Assay
Nanovectors	5K-M_sat_ (emu g^−1^)	Diamagnetic content (organic coating) (wt%)	TRAIL content (mol./part.)	Weight loss (wt%)	TRAIL content (mol./part.)	TRAIL content (mol./part.)
NH-TRAIL@NPs-CO	65 ± 4 (*)	22.8	15 ± 3	15.8	12 ± 4	13 ± 3
CO-TRAIL@NPs-NH	71 ± 5 (*)	15.7	11 ± 2	13.0	10 ± 3	10 ± 2

## Supplementary Materials

The following are available online at https://www.mdpi.com/2079-4991/11/2/502/s1, Figure S1. (a) NPs-CO and NPs-NH size distribution as inferred from TEM observations and (b) DLS measurements. (c) Zeta potential measured as a function of pH on aqueous suspensions of bare NPs and their hybrids; Figure S2. TG analysis of NPs-NH and CO-TRAIL@NPs-NH (right) compared with that of NPs-CO and NH-TRAIL@NPs-CO (left). The weight losses related to physisorbed water and organic coating departures are indicated by the black arrows; Table S1: SASA, in Å^2^ per residue, and for the carboxylic acid or amino groups of residues able to form an amide bond with either silane- or citrate-functionalized groups. The RMSF values are in Å.
