# Supplementary material for: Grafting TRAIL through Either Amino or Carboxylic Groups onto Maghemite Nanoparticles: Influence on Pro-Apoptotic Efficiency
Source: Nanomaterials (Basel). 2021 Feb 17;11(2):502. doi: 10.3390/nano11020502 (PMC7922020; doi:10.3390/nano11020502)
Supplement: Supplementary file 1 [file nanomaterials-11-00502-s001.pdf]

nanomaterials

Supporting Information

Grafting TRAIL through either Amino or Carboxylic Groups onto Maghemite Nanoparticles: Influence on Pro-Apoptotic Efficiency

Hanene Belkahla [1,2,3], Andrei Alexandru Constantinescu [2], Tijani Gharbi [3], Florent Barbault [1], Alexandre Chevillot [1], Philippe Decorse [1], Olivier Micheau [2], Miryana Hémadi [1,*,†] and Souad Ammar [1,*,†]

1 Université de Paris, CNRS-UMR 7086, Interfaces, Traitements, Organisation et DYnamique des Systèmes (ITODYS), Paris, France; hanen_belkahla@hotmail.fr (H.B.); florent.barbault@univ-paris-diderot.fr (F.B.); alexandre.chevillot@univ-paris-diderot.fr (A.C.); philippe.decorse@univ-paris-diderot.fr (P.D.)
2 LNC, Université de Bourgogne Franche-Comté, INSERM-UMR 1231, Dijon, France; andrei.ac@windowslive.com (A.A.C.); olivier.micheau@inserm.fr (O.M.)
3 Nanomedicine Lab, EA 4662, Université de Bourgogne Franche-Comté, Besançon, France; tijani.gharbi@me.com
* Correspondence: hemadi@univ-paris-diderot.fr (M.H.); ammarmer@univ-paris-diderot.fr (S.A.)
† Contributed equally to this work.

Citation: Belkahla, H.; Constantinescu, A.A.; Gharbi, T.; Barbault, F.; Chevillot, A.; Decorse, P.; Micheau, O.; Hémadi, M.; Ammar, S. Grafting TRAIL through Either Amino or Carboxylic Groups onto Maghemite Nanoparticles: Influence on Pro-Apoptotic Efficiency. *Nanomaterials* **2021**, *11*, 502. https://doi.org/10.3390/nano11020502

Academic Editor: Jose L. Luque-Garcia

Received: 1 February 2021
Accepted: 16 February 2021
Published: 17 February 2021

Publisher's Note: MDPI stays neutral with regard to jurisdictional claims in published maps and institutional affiliations.

Copyright: © 2021 by the authors. Licensee MDPI, Basel, Switzerland. This article is an open access article distributed under the terms and conditions of the Creative Commons Attribution (CC BY) license (http://creativecommons.org/licenses/by/4.0/).

Abstract: Tumor necrosis factor (TNF)-related apoptosis-inducing ligand (TRAIL) is a member of the TNF cytokine superfamily. TRAIL is able to induce apoptosis through engagement of its death receptors DR4 and DR5 in a wide variety of tumor cells while sparing vital normal cells. This makes it a promising agent for cancer therapy. Here, we present two different ways of covalently grafting TRAIL onto maghemite nanoparticles (NPs): (a) by using carboxylic acid groups of the protein to graft it onto maghemite NPs previously functionalized with amino groups, and (b) by using the amino functions of the protein to graft it onto NPs functionalized with carboxylic acid groups. The two resulting nanovectors, NH-TRAIL@NPs-CO and CO-TRAIL@NPs-NH, were thoroughly characterized. Biological studies performed on human breast and lung carcinoma cells (MDA-MB-231 and H1703 cell lines) established these nanovectors are potential agents for cancer therapy. The pro-apoptotic effect is somewhat greater for CO-TRAIL@NPs-NH than NH-TRAIL@NPs-CO, as evidenced by viability studies and apoptosis analysis. A computational study indicated that regardless of whether TRAIL is attached to NPs through an acid or an amino group, DR4 recognition is not affected in either case.

Keywords: surface functionalization; maghemite; cell viability; cancer disease; molecular modeling

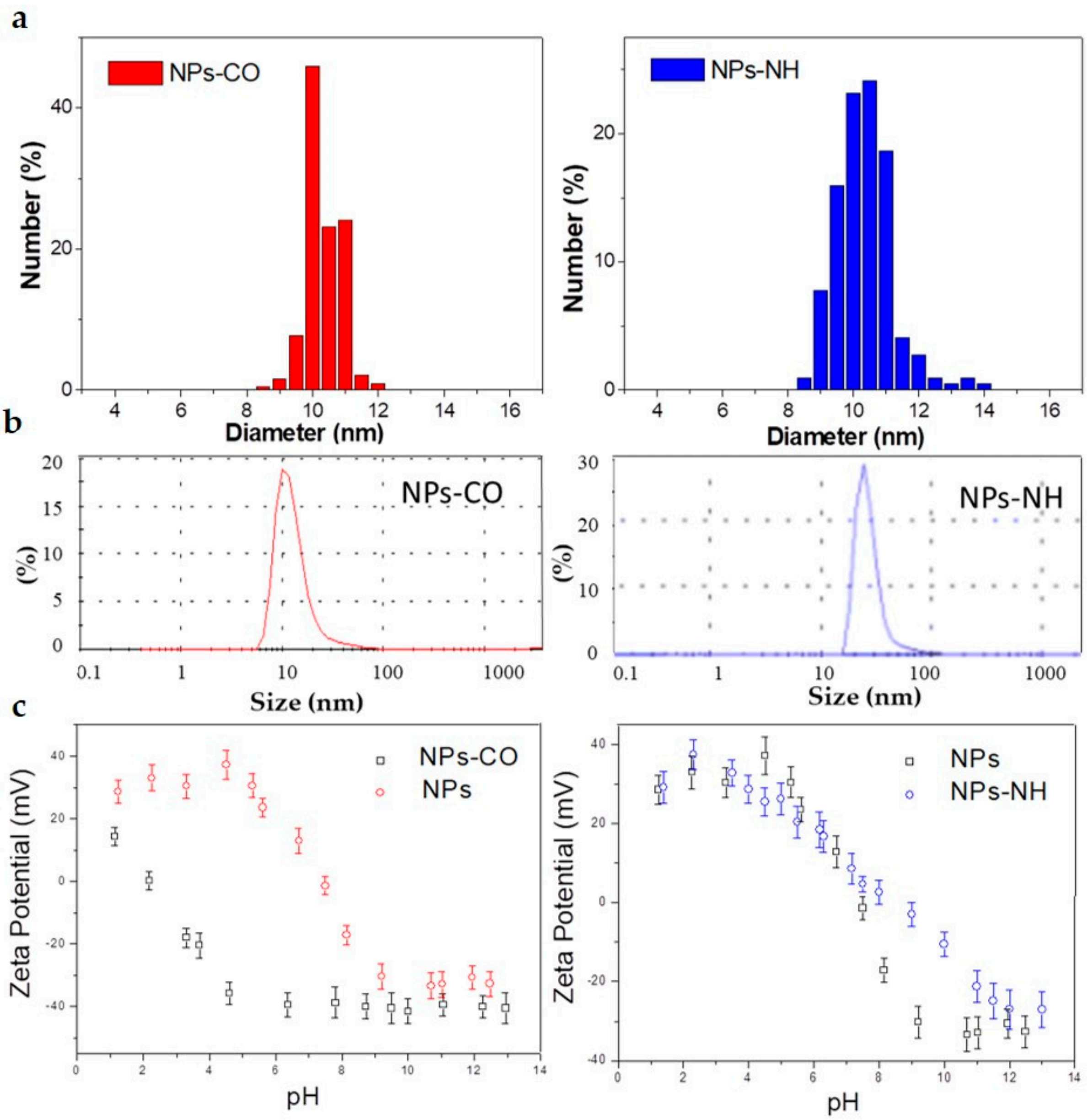

Figure S1. (**a**) NPs-CO and NPs-NH size distribution as inferred from TEM observations and (**b**) DLS measurements. (**c**) Zeta potential measured as a function of pH on aqueous suspensions of bare NPs and their hybrids.

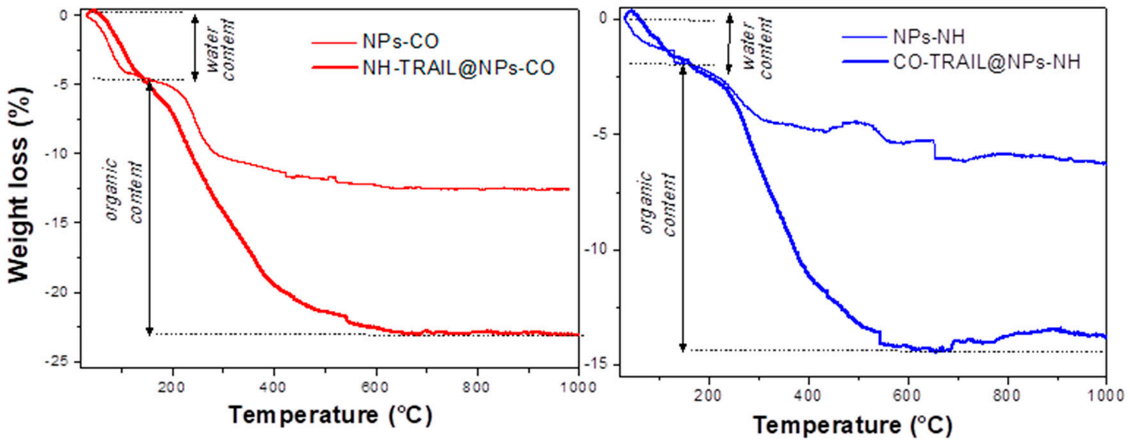

Figure S2. TG analysis of NPs-NH and CO-TRAIL@NPs-NH (right) compared with that of NPs-CO and NH-TRAIL@NPs-CO (left). The weight losses related to physisorbed water and organic coating departures are indicated by the black arrows.

Table S1. SASA, in Å² per residue, and for the carboxylic acid or amino groups of residues able to form an amide bond with either silane- or citrate-functionalized groups. The RMSF values are in Å.

	Acid for APTES bonding				Amino for acid bonding		
**Heading**	**SASA Res.**	**SASA COO-**	**RMSF**	**Heading**	**SASA Res.**	**SASA NH3+**	**RMSF**
**Glu4**	313.26	173.11	19.96	**Met1**	320.48	129.20	18.29
**Asp122**	278.63	169.79	16.33	**Lys67**	300.71	128.90	16.48
**Glu83**	300.64	164.61	15.09	**Lys85**	320.32	126.22	16.63
**Glu66**	318.71	163.78	18.11	**Lys121**	321.58	122.85	16.67
**Glu140**	303.06	162.20	18.21	**Lys139**	292.38	116.65	17.14
**Glu124**	311.20	161.44	14.73	**Lys38**	292.14	114.89	11.07
**Glu43**	301.98	154.09	11.64	**Lys30**	292.04	114.23	15.56
**Glu159**	283.40	153.86	9.32	**Lys33**	302.16	113.81	13.24
**Asp157**	268.53	153.51	9.00	**Lys112**	289.04	112.73	9.42
**Glu86**	293.04	153.25	17.84	**Lys100**	285.71	112.42	13.18
**Asp155**	268.39	152.60	8.81	**Lys89**	282.01	111.64	14.17
**Asp142**	261.77	152.28	15.95	**Lys92**	276.23	109.18	10.44
**Glu82**	280.13	151.85	14.04				
**Gly169**	207.60	148.72	17.80				
**Asp91**	237.18	147.56	11.11				
**Glu151**	285.82	146.33	10.23				
**Asp106**	221.26	146.33	15.24				
**Glu61**	282.15	145.98	13.20				
**Glu137**	267.40	145.37	15.40				
**Glu32**	269.47	143.52	13.36				
